# Effects of cognitive impairment and functional limitation on depressive symptoms among community-dwelling older Korean immigrants in the U.S.

**DOI:** 10.1371/journal.pone.0193092

**Published:** 2018-03-26

**Authors:** Bum Jung Kim, Lin Liu, Christabel Cheung, Joonhee Ahn

**Affiliations:** 1 Department of Social Welfare, Chung-Ang University, Seoul, South Korea; 2 Department of Sociology, University of Hawaii at Manoa, Honolulu, Hawaii, United States of America; 3 School of Social Work, University of Hawaii at Manoa, Honolulu, Hawaii, United States of America; 4 Department of Social Welfare, Hoseo University, Chunan-city, Chungchungnam-do, South Korea; Banner Alzheimer’s Institute, UNITED STATES

## Abstract

The present study examined the effects of cognitive impairment and functional limitation on depressive symptoms among older Korean American immigrants. The sample was drawn from a cross-sectional survey of 210 older Korean immigrants (aged 65 years or older) in Los Angeles County. Based on robust hierarchical regression, the study found that cognitive ability and functional status were significant explanatory factors related to depressive symptoms among older Korean immigrants. In addition, the interaction of instrumental activities of daily living (IADL) and cognitive function (MMSE) had a significant effect on depressive symptoms. This finding suggests that older Korean immigrants in the U.S. who experience deficits in cognitive function and/or IADL performance are vulnerable to psychological distress as indicated by depressive symptoms. Recommendations include implementing culturally-responsive health interventions aimed at enabling accessibility to dementia care services and supporting improvement of IADL performance among older Korean American immigrants.

## Introduction

Older Korean immigrants in the U.S. are the fifth largest Asian American ethnic subgroup [1]. In the three decades between 1970 and 2000, the number of older Korean American individuals increased twenty-fold, from 3,270 to 66,254 [1]. For this rapidly growing population, depression has been identified as a major mental health problem. Studies indicate that depressive symptoms are up to four times more prevalent in older Korean Americans than in the general older population [2]. Moreover, the rate of depression among older Korean immigrants is estimated at twice the rates of depression found among older Filipino, Chinese, and Japanese Americans, respectively [3]. In response to these troubling trends, previous research has identified multiple risk factors (e.g., poor health status, low socioeconomic status, acculturative stress and limited personal resources) associated with depressive symptoms in this high-risk group [4–6]. Findings from these studies indicate that impairment in cognitive ability is closely linked to depressive symptoms among community-dwelling older Korean immigrants [4, 7–8]. However, less is known about how cognitive impairment relates to other physical risk factors, thereby possibly exacerbating the risk of depression.

It is well-documented that cognitive impairment is comorbid with late-life depression, which is associated with decreased quality of life [9], greater health care utilization, and higher mortality rates [10–13]. These serious health and psychosocial consequences point to the need for developing strategies aimed at prevention, early detection, and intervention of depressive symptoms in older adults with cognitive impairment. Expanding geriatric knowledge regarding the relationship between cognitive impairment and comorbid depressive symptoms is critical to the implementation of effective prevention and treatment.

While some studies have examined the association between cognitive impairment and late-life depression, few have investigated the influence of functional ability on depression among older adults. For many older adults, cognitive impairment is often accompanied by functional disability, a diminished capacity to perform basic daily activities required for independent living in the community [14]. For older adults with cognitive impairment, the co-occurrence of functional limitations exacerbates the severity of strain, as this dual challenge presents serious ongoing issues with managing instrumental activities of daily living and sustaining essential social relationships [15]. In other words, the presence of functional limitations may increase the risk of depression for older adults with cognitive impairment.

Converging evidence supports the idea that cognitive impairment affects functional limitation and depression for the general population of older adults. Few investigations have explored whether cognitive impairment and functional limitations interactively influence depression. Notably, Kiosses and Alexopoulos [16] found that cognitive impairment assessed by the mini-mental status examination (MMSE) had a significant effect on the presence of depressive symptoms and functional limitation in instrumental activities of daily living (IADL) among elderly patients.

The present study examines how cognitive impairment, functional limitation in instrumental activities of daily living (IADL), and their interaction affect depressive symptoms among community-dwelling older Korean immigrants in the U.S. Given the heightened vulnerability for depression among this population, the results of this study will generate empirical evidence that will be helpful to developers of interventions and treatments aimed at promoting the mental health and well-being of older Korean American immigrants.

## Methods

### Sampling and participants

The study sample was drawn from a survey of community-dwelling older Korean immigrants (ages 65 years or older) in Los Angeles County in 2010. Due to limited availability of sampling units to constitute the study population, convenience sampling was used for participant recruitment. Data were collected at local Korean churches, adult day health care centers, senior centers, and senior associations. Survey questionnaires were provided in Korean using back translation, with the surveys first drafted in English, and then translated into Korean by professional translators. Trained bilingual social workers conducted face-to-face interviews in Korean and each interview took approximately 40 minutes. Face-to-face interviews were advantageous in ensuring high response rates and allowing for the respondents’ interpretations of questions to be probed and clarified [17]. The screening process contained the administration of the Short Portable Mental Status Questionnaire (SPMSQ) to verify participants’ capacity for study participation. Only participants with scores of 8 or higher were included in the survey. All study participants gave informed consent before their inclusion in this study. After obtaining IRB approval from the UCLA Institutional Review Board, data were collected at sites that were conveniently located to study participants, e.g. counseling room in service agencies, cafeteria in churches, and various resting areas. Upon completion of each interview, each participant was compensated with $20. The resulting sample included 210 participants. There were no missing data, and all participants were included in the data analysis.

### Measures

#### Geriatric Depression Scale

Extensively used with older adults in community, acute, and long-term care settings, the 15-item Geriatric Depression Scale-Short Form (GDS-SF) was administered orally to measure depressive symptoms [18]. The GDS-SF is solicits yes/no responses in five positively-framed questions (e.g. “Are you satisfied with your life?” and “Do you feel happy?”) and 10 negatively-framed questions (e.g. “Do you feel that your life is empty?” and “Do you feel helpless?”). The total GDS-SF score was calculated by tallying the number of “yes” responses to the negatively-framed questions, and reversing scores for the positively-framed questions. Total scores range from 0 to 15, with scores of 11 or greater indicating possible depression. Previous studies have translated the GDS-SF into Korean, and validated its psychometric properties [18]. The internal reliability for the GDS-SF in this study was .81.

#### Cognitive function (MMSE)

Assessment of each participant’s orientation to time and place, recall ability, short-term memory, and arithmetic ability was based on the mini-mental status examination (MMSE). The MMSE includes 11 items, divided into two sections. The first section requires verbal responses to orientation, memory, and attention questions. The second section captures reading, writing, and the ability to name items and follow verbal and written commands. Questions are asked in the order listed, and can be scored immediately by summing the points assigned to each successfully competed task. The maximum score is 30, and the most commonly used cut-point to indicate cognitive impairment is 23/24 or below [19–20]. The MMSE has high test-retest and inter-rater reliability. It correlates well with tests of functional ability such as ADL and IADL [21–22]. In the current study, Cronbach’s alpha for the MMSE scale was .76.

#### Instrumental activities of daily living (IADL*)*

Functional limitation was measured by the 10-item instrumental activities of daily living (IADL) scale, which was adapted from the Older Americans Resources and Services Multidimensional Functional Assessment Questionnaire [23]. Within this scale, questions about “instrumental” activities include the ability to use the telephone, shop for food, prepare meals, keep house, do laundry, use public transportation or drive, and manage personal finances. Items are scored on a two-point scale from 0 to 1, in which 0 indicates complete limitation with respect to a specific task. The current study utilized the Korean version of the IADL (K-IADL), and its Cronbach’s alpha for internal consistency was .81.

#### Demographic variables

Demographic information collected for participants included age (in years), gender (female = 0, male = 1), marital status (single = 0, married = 1; single included not married, divorced, widowed), education (no education, elementary, middle, high, college, graduate), and income.

### Statistical analyses

Data were analyzed using the software package STATA 13.0. Descriptive statistics and correlational analyses were conducted to show basic characteristics within the sample in addition to relationships among study variables. In particular, the study assessed variance inflation factors to check for multicollinearity. Researchers confirmed that variance inflation factor for all measures was less than 2.8. Hierarchical regression analyses of depressive symptoms was conducted in four sets: 1) demographic variables, 2) instrumental activities of daily living (IADL), 3) cognitive function (MMSE), and 4) interaction term between IADL and MMSE.

## Results

### Characteristics of the sample

Table 1 summarizes descriptive information related to the sample and study variables. The total study sample was 210 older Korean immigrants. Age among participants ranged from 65 to 98 years with an average of 81.36. Females comprised 70% versus 30% of males. Approximately 60% of participants were not married, and 40% were married at the time of survey. In terms of educational level, 11% indicated no education followed by 32% elementary, 11% middle school, 32% high school, 8% college, and 3.3% graduate school. Average monthly income was $1,142.83 (SD = 465.63) with a range of zero income to $4,500 per month. MMSE scores ranged between 15 to 30 with an average of 25.07 (SD = 3.41). Approximately 29% of the study participants are at risk of being diagnosed with dementia according to the cutoff score of 24 or below on the MMSE. The average score of IADL was 4.92 (SD = 1.90) with a range from 0 to 8. The mean score of depressive symptoms was 3.46 (SD = 2.43, range = 0–10). None of the study participants were at risk of being diagnosed with depressive symptoms, using a cutoff score of 11 or greater to indicate possible depressive symptoms.

**Table 1:** Descriptive statistics of older Korean immigrants (N = 210).

Variable	N	%
Age		
-Range	65–98	
-Mean (SD)	81.36 (6.98)	
Gender		
-Female	147	70.00
-Male	63	30.00
Marital Status		
- Not married	123	58.57
- Married	87	41.43
Education		
-None	24	11.43
-Elementary	69	32.86
-Middle	24	11.43
-High	68	32.38
-College	18	8.57
-Graduate	7	3.33
Income		
-Range	0–4500	
-Mean (SD)	1142.83 (465.63)	
Cognitive function (MMSE)		
-Range	15–30	
-Mean (SD)	25.07 (3.41)	
IADL		
-Range	0–8	
-Mean (SD)	4.92 (1.90)	
Depression		
- Range	0–10	
- Mean (SD)	3.46 (2.43)	

### Correlation

Using variance inflation factor analysis, the study confirmed the absence of multicollinearity (VIF values < 5) among study variables. The study analyzed bivariate correlations among the study variables before conducting multivariate modeling. Table 2 shows that the variables were correlated in the expected direction and found no multicollinearity issues. The highest coefficient was found in the relationship between depressive symptoms and gender (r = -.37, p <.01). Similarly, the coefficient for the relationship between depressive symptoms and cognitive function (r = -.18, p <.01) was high, and that of depressive symptoms and IADL (r = -.15, p <.05) was at a moderate level.

**Table 2:** Correlations among study variables.

	1	2	3	4	5	6	7
1. Age	-						
2. Gender	.02						
3. Marital Status	-.22**	.31**					
4. Education	-.23**	.28**	.12				
5. Income	.06	.20**	.11	.08			
6. IADL	-.08	.00	.18**	-.08	.09		
7. Cognitive Function	-.18**	.21**	.19**	.37**	.25**	.16*	
8. Depression	-.30**	-.37**	-.25**	-.00	-.09	-.15*	-.18**

* p <.05.

** p <.01.

Gender: Female = 0, Male = 1. Marital Status: Single = 0, Married = 1

### Hierarchical regression

Table 3 summarizes results of the hierarchical regression analyses for depressive symptoms. Altogether, the set of demographic variables included in this analysis explained 24% of the total variance of depressive symptoms for the study sample. Participants who were younger, female, and single were more likely to be depressed. The second model added IADL and explained an additional 3% of the variance. Participants with lower IADL were more likely to be depressed and IADL was found to be a significant predictor of depressive symptoms. With the addition of cognitive function (MMSE) in the third model, an additional 2% of the variance was explained, confirming that cognitive function was negatively associated with depressive symptoms. As a final step, an interaction term of IADL and MMSE was entered, producing a model that added another 2% of the variance.

**Table 3:** Hierarchical regression models on depression.

Variables	Model 1		Model 2		Model 3		Model 4	
	Beta	T value	Beta	T value	Beta	T value	Beta	T value
Age	-.34	-6.17**	-.35	-5.83**	-.37	-5.91**	-.34	-5.69**
Male	-.29	-4.65**	-.29	-4.91**	-.28	-4.62**	-.26	-4.35**
Married	-.24	-3.70**	-.21	-3.33**	-.20	-3.06**	-.21	-3.21**
Education	.03	.54	.01	.22	.06	1.11	.07	1.24
IADL			-.14	-2.73**	-.11	-2.20*	-.49	-2.09*
Cognitive Function					-.16	-2.38*	-.43	-2.32*
*IADL x Cognitive Function*							.48	-1.63*
R^2^	.27		.29		.31		.33	
R^2^ change			.02**		.02**		.01*	
Adjusted R^2^	.24		.27		.29		.31	
F	20.04**		17.16**		14.60**		12.46**	

* p <.05.

** p <.01.

Gender: Female = 0, Male = 1. Marital Status: Single = 0, Married = 1

**Fig 1:**
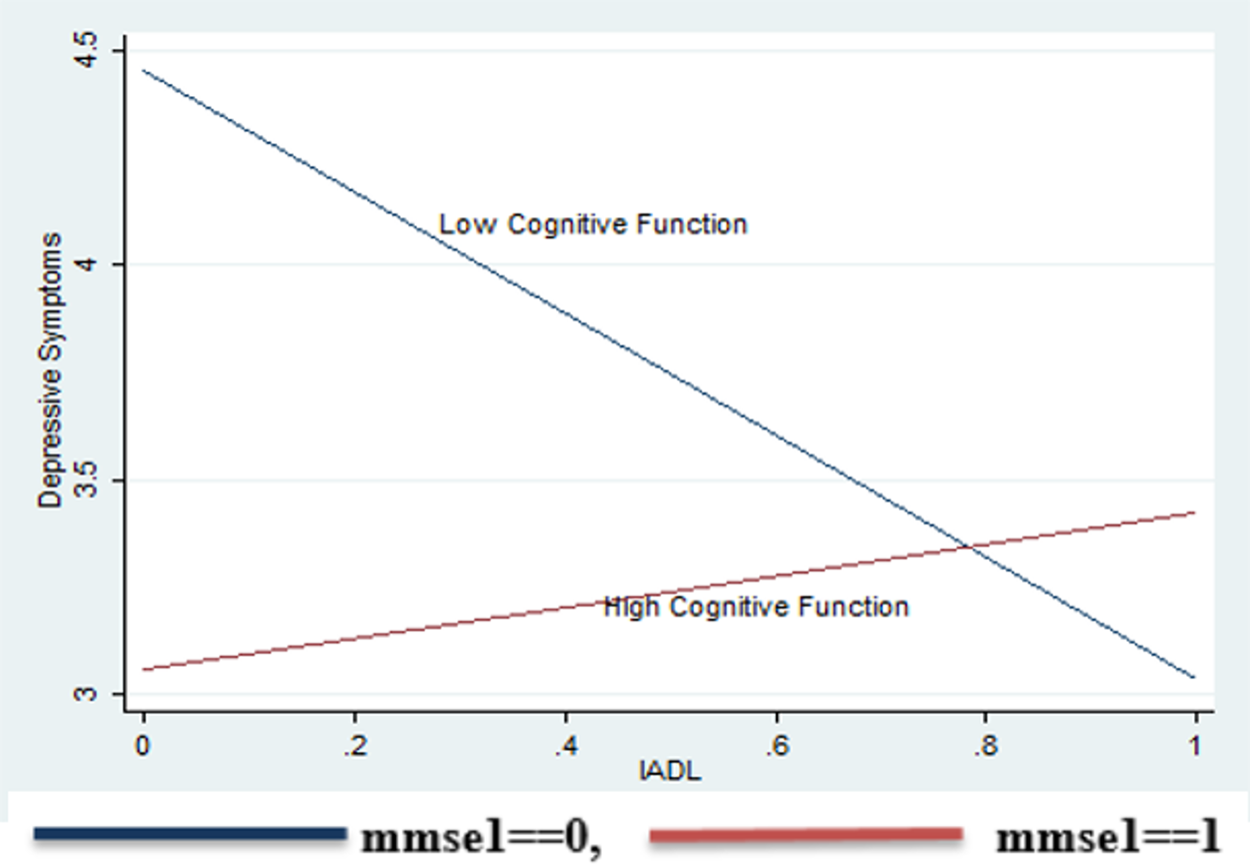
Interaction effect of IADL and cognitive function (MMSE) on depressive symptoms.

For further investigation of the interaction effect (Fig 1), we divided the sample into two subgroups, one with low cognitive function (below average MMSE scores, n = 99) and another with high cognitive function (n = 111), and compared their correlations between IADL and depressive symptoms. In the group with high MMSE, the association between IADL and depressive symptoms was low and not significant (r = .0.6, p>.05). On the contrary, the association between IADL and depressive symptoms was strong and negatively significant in the subgroup with low MMSE (r = -.30, p <.01). The difference in the bivariate coefficients between these two groups was significant (t = 7.23, p <.01).

## Discussion and conclusions

The present study examined the effects of cognitive impairment and functional limitations on depressive symptoms among older Korean immigrants, after controlling for the effects of demographic variables. This investigation further examined the influence of the interaction effect of functional limitations in IADL and cognitive function (MMSE) on depressive symptoms. Findings revealed that depressive symptoms were significantly associated with both functional limitation and cognitive impairment within the study population.

Evidence derived from the current study domonstrated that there is a significant negative relationship between functional limitation in IADL and depressive symptoms, which aligns with previous findings [3, 24]. These findings suggest that when older Korean immigrants exhibit high performanve on IADL tasks, they are able to enjoy greater independence and sustain more active and satisfying lives. Independent functioning included better management of personal finances, accurate medication management to maintain health, and the ability to make phone calls and maintain social ties as they wish. Independent telephone usage is particularly critical, because older adults with this capacity are able to stay connected with members of their social network such as family members, friends, and acquaintances, thereby increasing possibilities to enjoy private conversations that minimize feelings of isolation and loneliness [24].

Consistent with previous studies, one of the major findings of the present research was that cognitive impairment has a significant impact on depressive symptoms for older Korean immigrants in the U.S. In addition to finding significant independent effects for both functional limitation and cognitive ability in relation to depressive symptoms, the interaction term of functional limitation and cognitive impairment was also found to have a significant effect on depressive symptoms. To clarify this relationship, an additional analysis was performed, the results of which indicated that the correlations between MMSE and depressive symptoms were significantly stronger in the group with low levels of IADL function. More specifically, the significant effect of cognitive impairment on depression had a critical influence for those with low IADL functions. Older Koreans with both poor cognition and poor functional ability are more likely to experience the loss of practical day-to-day independence, which may put strains on their social relationships and lower their self-esteem [4, 25].

Insights from the present research include recommendations for implementing culturally-responsive health interventions aimed at enabling accessibility to dementia care services and supporting improvement of IADL performance among older Korean American immigrants. For example, community outreach programs should prioritize identifying high-risk older adults by employing bicultural case managers and health care professionals who can provide culturally-responsive practical assistance, and make appropriate relevant referrals to meet the needs of the individual. In parallel, community centers can host educational seminars that introduce trusted resources and creative strategies to address common challenges in the management of IADL and cognitive issues, e.g. medication and financial management [4, 8, 26]. Given the high rate of participation in religious organizations (95%) among older Korean immigrants, collaboration between local churches and community centers can enhance early detection of isolated older Koreans, and connect them to culturally relevant mental health services. Finally, government funding should be directed towards increasing the availability of in-home support services aimed at supporting cognitive impairment and IADL that builds upon existing strengths within the individual, family, and community contexts of older Korean immigrants [27, 28].

Notwithstanding its contributions, the present study had several limitations that should be acknowledged. Since the study is based on a cross-sectional survey design, causality among the study variables cannot be verified. For instance, it is equally possible that cognitive impairment is an outcome of depression. Future investigation should pursue longitudinal study that delves into the possible time-variant complexity of this linkage more closely. Another limitation is related to sampling. The average score for IADL performance in the current study sample is quite high, with almost half of participants (44%) having obtained more than high school education. As a consequence, the sample may be skewed towards participants of middle/upper socioeconomic class standing, with strong social support and greater physical capacity. Future studies should, therefore, improve the generalizability of results by including a wider cross-section of older Korean immigrants. Additionally, the study used shorter versions of both the cognitive screening test (SPMSQ) and the Geriatric Depression Scale (GDS); full-versions may have increased the sensitivity of participants’ assessments. Lastly, the study focused on depressive symptoms, and did not include other psychological disorders such as anxiety and hopelessness. Despite the aforementioned limitations, findings from the present study provide crucial empirical evidence regarding the effects of cognitive impairment and functional limitation on depression with practical implications for the promotion of culturally-responsive mental health programming for older Korean immigrants in the U.S.
